# THE UNINTENDED CONSEQUENCES OF SEAMLESS IMPLEMENTATION OF A RURAL TELEMEDICINE CLINIC

**DOI:** 10.1093/geroni/igz038.2263

**Published:** 2019-11-08

**Authors:** Samantha Solimeo, Melissa J Steffen, Aaron Seaman, Karla L Miller

**Affiliations:** 1 Center for Access and Delivery Research and Evaluation Primary Care Analytics Team, Iowa City VA Health Care System, Iowa City, Iowa, United States; 2 Veterans Rural Health Resource Center Central Region Center for Access and Delivery Research and Evaluation, Primary Care Analytics Team, Iowa City, Iowa, United States; 3 Carver College of Medicine, Iowa City, Iowa, United States; 4 Department of Internal Medicine, Rheumatology Section;, Salt Lake City, Utah, United States

## Abstract

Osteoporosis is largely undetected and untreated in older men who are at-risk, with even lower rates of detection and treatment in rural communities. In this presentation we report the consequences for primary care providers (PCPs) of the implementation of an osteoporosis telemedicine clinic that targeted rural patients and is purposefully designed to minimize PCP workload. Analysis of qualitative interviews with PCPs who co-managed patients with the telemedicine clinic confirmed that implementation strategies used by the telemedicine clinic were successful at minimizing workload and had a positive impact on PCPs’ work. However, the clinic’s seamless approach may have contributed to unintended consequences of low PCP awareness of program care processes and selection criteria and a missed opportunity for PCPs to reinforce osteoporosis care goals in the primary care setting.

